# Supplementary material for: A Comprehensive Genetic Study of Microtubule-Associated Gene Clusters for Male Infertility in a Taiwanese Cohort
Source: Int J Mol Sci. 2023 Oct 19;24(20):15363. doi: 10.3390/ijms242015363 (PMC10607339; doi:10.3390/ijms242015363)
Supplement: Supplementary file 1 [file ijms-24-15363-s001.zip › ijms-2612231-supplementary.pdf]

Full Title: A comprehensive genetic study of Microtubule-associated genes clusters for male infertility in a Taiwanese cohort

Short Title: Microtubule-associated genes clusters for male infertility

Chying-Chyuan Chan[1,2], Te-Hsin Yen[2], Hao-Chen Tseng[3], Brang Mai[3], Pin-Kuan Ho[4], Jian-Liang Chou[1,5], Gwo-Jang Wu[1,6*] and Yu-Chuan Huang[1,3,5*]

SUPPLEMENTARY TABLES

Table S1. Screening of Y chromosome microdeletion was used for testing samples for the presence of AZF-STS based on polymerase chain reaction. The map contains STSs in different AZF (Promega).

ID	Sample	Azoo S02	Oligo S06	Oilgo S07	Oligo S08	Azoo S09	Azoo S10	Oligo S11	Azoo S-2017
A Map	c18 (SY254)	■	□	□	□	□	□	□	▲
	c20 (SY157)	■	□	□	□	□	□	□	□
	a2 (SY81)	□	□	□	□	□	□	□	□
	b11 (SY130)	□	□	□	□	□	□	□	□
	b5 (SY182)	□	□	□	□	□	□	□	□
B Map	b7 (SYSYPR3)	□	□	□	□	□	□	□	□
	b9 (SY127)	□	□	□	□	□	□	□	□
	c16 (SY242)	■	□	□	□	□	□	□	□
	c17 (SY208)	■	□	□	□	□	□	□	□
C Map	b10 (SY128)	▲	□	□	▲	□	□	□	▲
	b6 (SY121)	□	□	□	□	□	□	□	□
	bc14 (145)	■	□	□	▲	□	□	□	□
	c19 (SY255)	■	□	□	□	□	□	□	□
D Map	b12 (SY133)	□	□	▲	□	□	□	▲	□
	c15 (SY152)	▲	□	□	□	□	□	□	□
	b8 (SY124)	■	□	□	□	□	□	□	□
E Map	b13 (SY134)	□	□	□	□	▲	□	▲	□
	a3 (SY86)	□	□	□	□	▲	□	▲	□
	a4 (SY84)	□	□	□	□	▲	□	▲	□

□: no deletion ▲: over 20% deletion ■: over 70% deletion

Table S2. The primers of SNPs on *ESR1*, *CFTR*, *SPATA16*, *STPG2*, *KIF6* and *SIMC1* were used to validate the targeted NGS and WES (upper table). The PCR reaction protocol is shown in the lower table and generally run for 34 cycles.

Gene	Forward primer	Reverse primer
STPG2	F:TGCAGGACATGTTGCTGTAG	R:AGACACGAACCACTTGACAG
KIF6	F: GATTACTATAAGCTAGCATC	R: ATCCATTACAGACAATGAG
ESR1	F: CTCAACAGCGTGTCTCCGAG	R: GAACTTGACTCTGAACGCAG
CFTR	F:GCAGAGTACCTGAAACAGGAAG	R:CTTTAATGGTGCCAGGCATA
SPATA16	F:TGTCCACCTTAGCGCACCCA	R:GGCTACCTGAAGCCATTTGTC
SIMC1	F:GAGATGTGTTACATTCACCT	R:CAGAGCTGAGATATCCATAG

Gene	Product Size	PCR				
			34 cycles (95°C 45sec → 72°C 30sec)			
SPATA16	405	95°C 5mins	95°C 45sec	58°C 45sec	72°C 30sec	72°C 5mins
CFTR	240					
			34 cycles (95°C 45sec → 72°C 30sec)			
GnRH1	256	95°C 5mins	95°C 45sec	54°C 45sec	72°C 30sec	72°C 5mins
ESR1	313					

Table S3. Variants identified and *in silico* evaluation of pathogenicity of nucleotide changes in exons of *CFTR* and *SPATA16* was performed using CADD, SIFT, Polyphen. MAF were checked in the GenomAD and Taiwan Biobank

Gene	Variant of gene and protein			Minor Allele Frequency% (genom AD)	Taiwan Biobank%	*In silico* programs		
	rs #	cDNA	encoded protein			CADD	SIFT	Polyphen 2
SPATA16	16846616	c.G440A	p.(G147E)	13.1	32.8	15.67	deleterious	Probably damaging
	1515442	c.A397G	p.(M133V)	30.99	39.3	19.12	deleterious	benign
	1515441	c.G232A	p.(E78K)	11.9	32.54	22.2	deleterious	possibly_damaging
CFTR	213950	c.G1408A	p.(V470M)	55.7	40.9	14.6	tolerated	benign

Minor allele frequency (MAF) in the Genome Aggregation Database gnomAD, TWB and TPMI in Taiwan (http://gnomad.broadinstitute.org/; https://taiwanview.twbiobank.org.tw/index)
Combined Annotation Dependent Depletion (CADD) (https://cadd.gs.washington.edu.)
Sorting Intolerant from Tolerant (SIFT, https://sift.bii.a-star.edu.sg/)
Polymorphism Phenotyping v2 (PolyPhen-2, http:// genetics.bwh.harvard.edu/pph2/)

Table S4. Variants identified and in silico evaluation of pathogenicity of nucleotide changes in exons of 15 candidate SNPs were performed using CADD, SIFT, Polyphen. MAF and MAF_eas were checked in the CLC module. The mutation frequencies in cases are compared to those in controls and are also provided in the list.

Gene	variant of gene and protein			MAF%	MAF_eas	in silico programs			p_value	cases_frequency	controls_frequency	gene_description
	rs#	cDNA	encoded protein			CADD	SIFT	Polyphen 2				
TPTE	1810856	G>A	R>Q	0.32	0.4	19.49	tolerated	possibly_damaging	1.40E-03	100	14.29	transmembrane phosphatase with tensin homology
CCDC168	1375719	A286G	I96V	0.41	0.4	0.053	tolerated	benign	0.03	100	42.86	coiled-coil domain 168
	12855785	T244A	C82S	0.41	0.4	4.786	tolerated	benign	0.03	100	42.86	
RADIL	3763384	G715A	D239N	0.25	0.37	8.017	tolerated	benign	0.03	100	42.86	Rap associated with DIL domain
TRIM49	12417980	G>A	G>R	0.3	0.36	6.251	tolerated	possibly_damaging	0.03	100	42.86	tripartite motif containing 49
KIF6	2273063	G1535A	R512H	0.04	0.25	9.791	tolerated	benign	1.24E-03	88	0	kinesin family member 6
STPG2	17558193	T373C	Y125H	0.39	0.3	25.6	deleterious	probably_damaging	8.86E-03	88	14.29	sperm tail PG-rich repeat containing 2
	2903150	A532G	I178V	0.39	0.3	5.46	tolerated	benign	8.86E-03	88	14.29	
DNAH2	7213894	C>T	T>I	0.63	0.34	14.29	tolerated	benign	0.03	88	28.57	dynein axonemal heavy chain 2
DNAH11	6461613	A>G	M>V	0.69	0.69	18.94	tolerated	benign	0.03	88	28.57	dynein axonemal heavy chain 11
DRC7	3809611	C>T	P>L	0.24	0.16	21.3	deleterious	benign	5.59E-03	75	0	dynein regulatory complex subunit 7
NEK2	2230489	G>A	G>R	0.16	0.22	16.01	tolerated	benign	0.03	75	14.29	NIMA related kinase 2
SART3	2072579	C69G;	D23E	0.2	0.34	9.179	tolerated	benign	0.03	75	14.29	spliceosome associated factor 3
DYNC2H1	10895391	C>T	A>V	0.3	0.28	23.7	tolerated	benign	0.03	75	14.29	dynein cytoplasmic 2 heavy chain 1
CMTM2	2290182	T365C	I122T	0.17	0.28	13.22	tolerated	benign	0.03	75	14.29	CKLF like MARVEL transmembrane domain containing 2
CATSPER2	8042868	G>A	V>I	0.15	0.28	13.79	deleterious	benign	0.03	75	14.29	cation channel sperm associated 2
BORCS5	3751262	G>A	D>N	0.06	0.2	29.9	deleterious	possibly_damaging	0.03	75	14.29	BLOC-1 related complex subunit 5

Table S5A**.** The designed amplicons (green) covered the *SPATA16* (chr3 q26.31) and *CFTR* (chr7 q31.2) regions of a 15 - gene targeted NGS panel (blue).

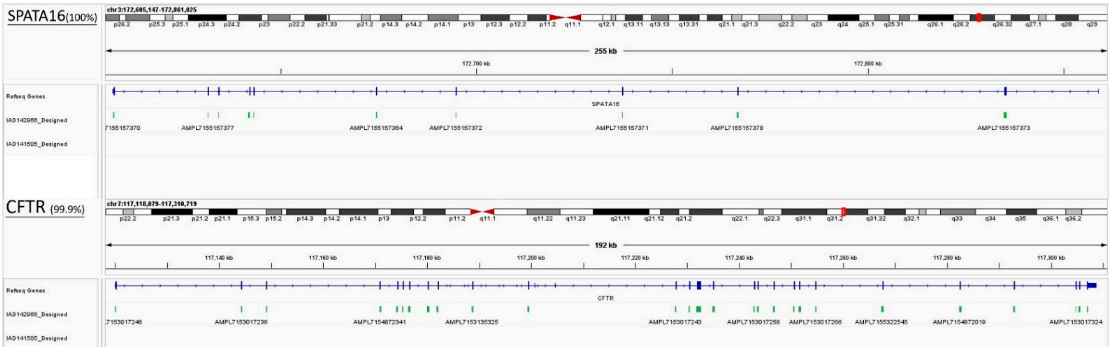

Table S5B**.** The designed amplicons (green) covered the *FSHB* (chr11 p14.1), *AURKC* (chr19q13.43), and *PICK1* (chr22q13.1) regions of a 15 - gene targeted NGS panel (blue).

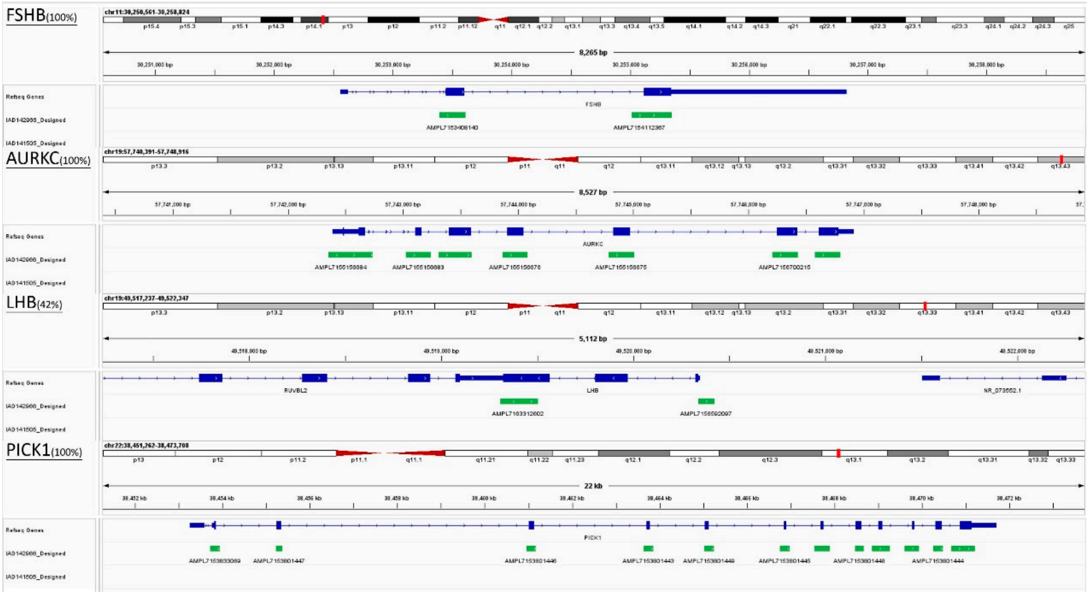

Table S5C**.** The designed amplicons (green) covered the *DAZL* (chr3 p25.1), *ESR1*(chr6 q25.1), *GnRH1*(chr25 p21.2), NR5A1(chr9 q33.3), and *FGFR1*(chr8 p11.23) regions of a 15 - gene targeted NGS panel (blue).

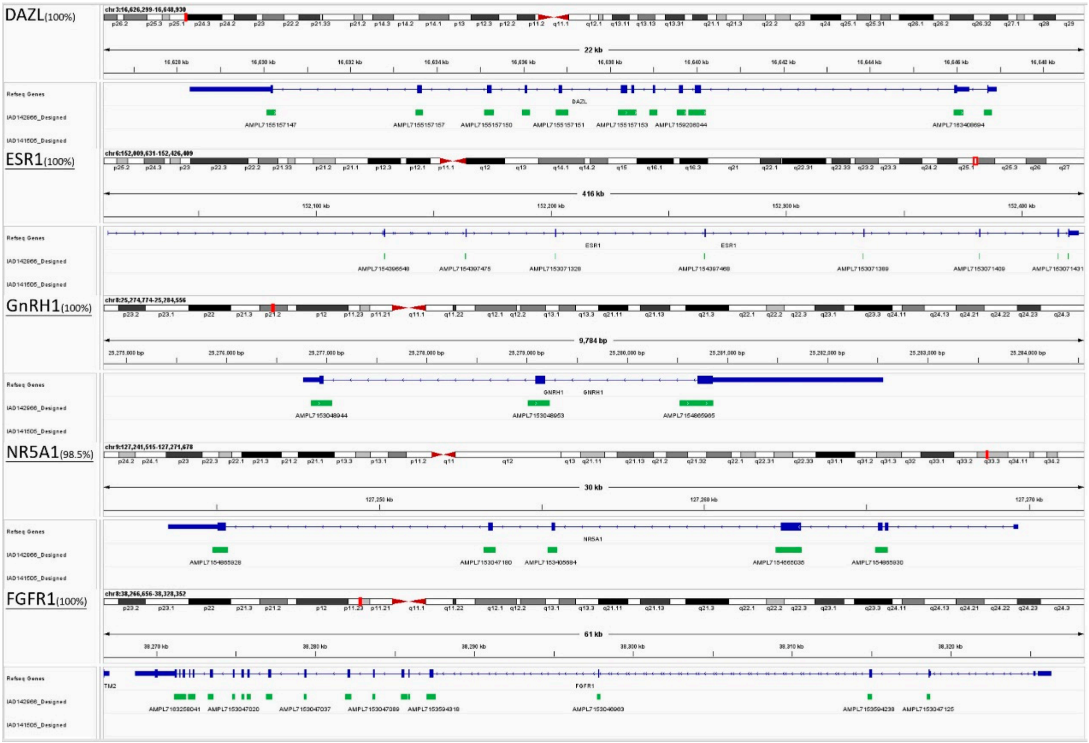

Table S5D**.** The designed amplicons (green) covered the *AR* (chrX q12), *TEX11*(chrX q13.1), *USP26* (chrX q26.1), and *ANOS1*(chrX p22.32) regions of a 15 - gene targeted NGS panel (blue).

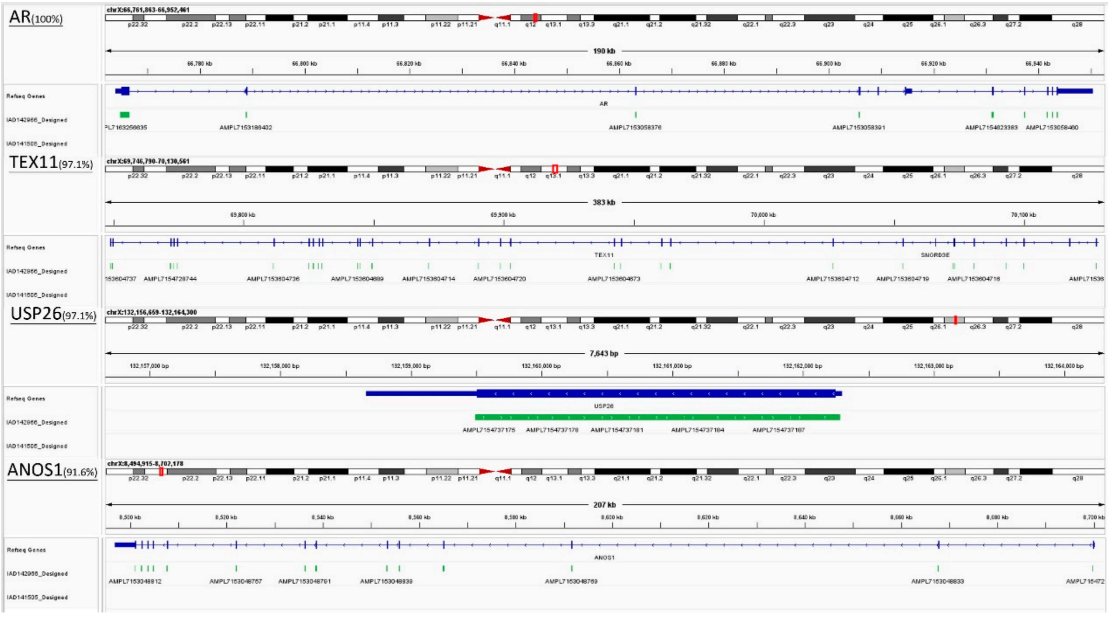

SUPPLEMENTARY FIGURES

Figure S1 AZF-STS based multiplex polymerase chain reaction (PCR) is routinely used to detect YCMD by tracing sequence-tagged sites (STSs) marks in the AZF (azoospermia factor region) at the long arm of Y chromosome (Yp) (Promega).

Figure S2 Screening of Y chromosome microdeletion (Promega) was used for testing samples for the presence of AZF-STS based on polymerase chain reaction. (red-labeled ones are represented azoospermia; black-labeled ones are represented oligozoospermia.

Figure S3 Identified SNP of KIF6 and STPG2 were confirmed by Sanger sequencing.

	SY #	DYS	AZF region
1	SY14	SRY	SRY
a2	SY81	DYS271	
a3	SY86	DYS148	AZFa
a4	SY84	DYS273	
b5	SY182	KALY	
b6	SY121	DYS212	
b7	SYPR3	SMCY	
b8	SY124	DYS215	
b9	SY127	DYS218	
b10	SY128	DYS219	AZFb
b11	SY130	DYS221	
b12	SY133	DYS223	
b13	SY134	DYS224	
b14	SY145	DYF51S1	
b15	SY152	DYS236	
c16	SY242	DAZ	
c17	SY208	DAZ	AZFc
c18	SY254	DAZ	
c19	SY255	DAZ	
c20	SY157	DYS240	

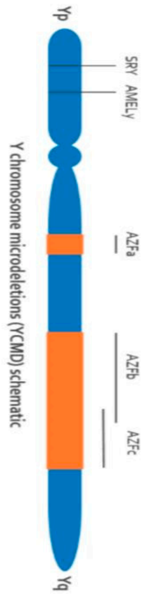

Figure S1

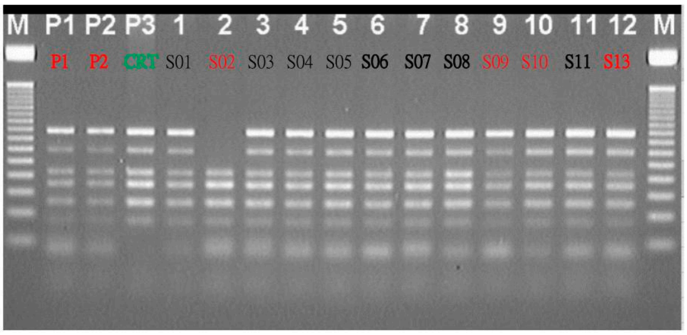

Figure S2

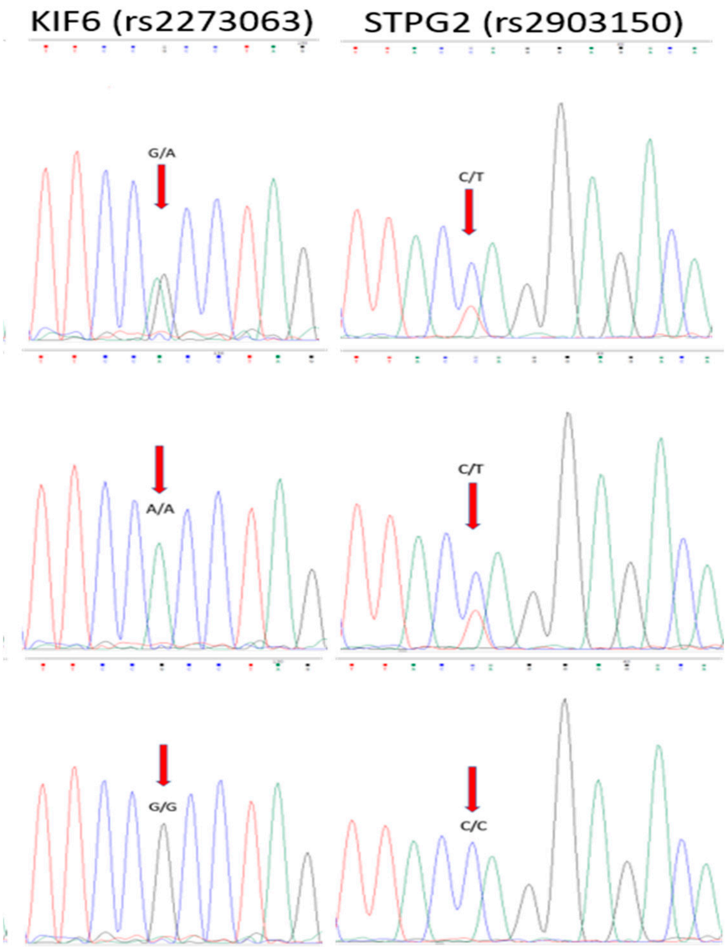

Figure S3
